# Improvement of *Agrobacterium*-mediated transformation of cucumber (*Cucumis sativus* L.) by combination of vacuum infiltration and co-cultivation on filter paper wicks

**DOI:** 10.1007/s11816-012-0260-1

**Published:** 2012-09-18

**Authors:** Yoshihiko Nanasato, Ken-ichi Konagaya, Ayako Okuzaki, Mai Tsuda, Yutaka Tabei

**Affiliations:** 1 Genetically Modified Organism Research Center, National Institute of Agrobiological Sciences, 2-1-2 Kannondai, Tsukuba, Ibaraki 305-8602 Japan; 2 Present Address: Forest Bio-Research Center, Forestry and Forest Products Research Institute, 3809-1 Ishi, Juo, Hitachi, Ibaraki 319-1301 Japan

**Keywords:** Acetosyringone, *Agrobacterium*, *Cucumis sativus*, Filter paper wicks, Transformation, Vacuum infiltration

## Abstract

An improved method for genetic transformation of cucumber (*Cucumis sativus* L. cv. Shinhokusei No. 1) was developed. Vacuum infiltration of cotyledonary explants with *Agrobacterium* suspension enhanced the efficiency of *Agrobacterium* infection in the proximal regions of explants. Co-cultivation on filter paper wicks suppressed necrosis of explants, leading to increased regeneration efficiency. Putative transgenic plants were screened by kanamycin resistance and green fluorescent protein (GFP) fluorescence, and integration of the transgene into the cucumber genome was confirmed by genomic polymerase chain reaction (PCR) and Southern blotting. These transgenic plants grew normally and T_1_ seeds were obtained from 7 lines. Finally, stable integration and transmission of the transgene in T_1_ generations were confirmed by GFP fluorescence and genomic PCR. The average transgenic efficiency for producing cucumbers with our method was 11.9 ± 3.5 %, which is among the highest values reported until date using kanamycin as a selective agent.

**Electronic supplementary material:**

The online version of this article (doi:10.1007/s11816-012-0260-1) contains supplementary material, which is available to authorized users.

## Introduction

Cucumber (*Cucumis sativus* L.) is one of the most important vegetables in the world. Global production of cucumbers, including of gherkins, reached 60.6 million tons in 2009 (http://faostat.fao.org), which was among the top ten vegetables produced globally in 2009. Cucumber production is subject to infections by a wide range of pathogens including leaf fungal diseases, *Fusarium* wilt symptoms, nematode infestation, anthracnose, scab, leaf blotch, and virus diseases. However, it is difficult to improve the tolerance of cucumber to pathogen attack by conventional breeding owing to its narrow genetic base, with a genetic variability of only 3–8 % (Plader et al. 2007). Although grafting of cucumber plants onto cucurbitaceous rootstocks is a common way to avoid soilborne diseases and nematodes (Oda 2008), this technique is labor-intensive. Genetic engineering is a powerful way to improve biotic stress tolerance, because useful genes can be transferred from other species. Transgenic cucumber expressing a cucumber mosaic virus coat-protein gene showed virus resistance (Nishibayashi et al. 1996; Wako et al. 2001). A chitinase gene isolated from rice conferred effective resistance to *Botrytis cinerea* on cucumber (Tabei et al. 1998).

Trulson et al. (1986) first reported the transformation of cucumber via *Agrobacterium rhizogenes*. Transgenic cucumber plants were obtained via somatic embryogenesis from cotyledonary explants using *A*. *tumefaciens* (Chee 1990; Tabei et al. 1994). Spontaneous mutation is less frequent in direct organogenesis than in somatic embryogenesis; therefore, direct organogenesis is suitable for producing transgenic plants. Nishibayashi et al. (1996) succeeded in producing transgenic cucumber plants via direct organogenesis using hypocotyl explants with *A*. *tumefaciens*, and stem node explants were also used for transformation of cucumber via direct organogenesis (Miao et al. 2009). Several research groups have used cotyledonary explants for direct shoot organogenesis (He et al. 2006; Rajagopalan and Perl-Treves 2005; Tabei et al. 1998). Addition of abscisic acid (ABA) induced the efficient production of adventitious shoots from cotyledonary explants (Tabei et al. 1998). ABA treatment of cotyledonary explants for induction of organogenesis has been applied to a wide variety of cucumber cultivars (Gal-On et al. 2005; He et al. 2006; Rajagopalan and Perl-Treves 2005; Vengadesan et al. 2005).

Cucumber has received much attention as a model plant for Cucurbitaceae. In 2009, the genome sequence was decoded (Huang et al. 2009), and an integrated genetic and cytogenetic map of the cucumber genome was developed (Ren et al. 2009). The genomic database has been vastly developing (http://icugi.org). However, production of transgenic cucumber carrying agronomically important traits and reverse-genetic studies using transgenic cucumber have been infrequently reported. The reasons are thought to be that difficulties for the transformation of cucumber remained, although transformation of cucumber has been reported by several groups. Accordingly, we attempted to develop a more efficient and reproducible cucumber transformation via direct organogenesis. Recently, vacuum infiltration with *Agrobacterium* suspension succeeded in improving transformation efficiency of cowpea and citrus (Bakshi et al. 2011; de Oliveira et al. 2009), while use of filter paper wicks during the co-cultivation period also contributed to efficient transformation of pumpkin and rice (Nanasato et al. 2011; Ozawa 2009). In the present study, we investigated the combined effects of these treatments on cucumber transformation. In addition, we examined the effects of acetosyringone application to the co-cultivation medium and of the concentration of the *Agrobacterium* suspension on transformation efficiency.

## Materials and methods

### Preparation of cotyledonary explants

**Fig. 1:**
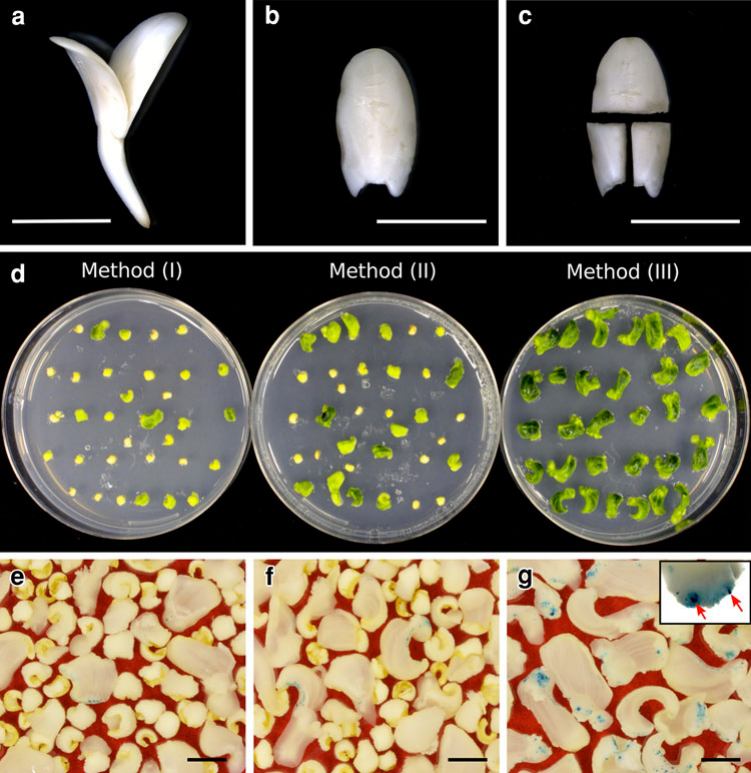
Preparation of explants from cotyledon of *C*. *sativus* cv. Shinhokusei No. 1 and effect of gel supports on *Agrobacterium* infection efficiency. **a** A maturing seed incubated for 1 day at 28 °C in the dark. *Bar* 5 mm. **b**, **c** A detached cotyledon from a maturing seed (**b**), and cut pieces of cotyledon (**c**). *Bars* 5 mm. **d** Cotyledonary explants incubated in various culture supports after *Agrobacterium* infection. Explants incubated with various gel supports for 1 week are shown. *Left to right* 0.8 % agar gel (method I), 0.8 % agar gel overlaid with a piece of filter paper (method II), and 3 filter papers moistened with 5.5 mL liquid medium (method III). **e**–**g** GUS activity in explants co-cultivated with method I (**e**), method II (**f**), and method III (**g**). The *inset* shows magnified GUS-positive cell clusters (*red arrows*) in the proximal region of the cotyledon explant. *Bars* 1 cm

Seeds of *C*. *sativus* (cv. Shinhokusei No. 1) were purchased from Tokiwa (Saitama, Japan). Cotyledonary explants were prepared as described previously (Tabei et al. 1998). Seed coats were removed with a scalpel and forceps. The peeled seeds were sterilized for 10 min using 1 % (w/v) sodium hypochlorite with 1 drop of Tween 20 and then rinsed 5 times with sterile distilled water. The sterilized seeds were germinated at 28 °C in the dark for 1 day in a plastic 9-cm Petri dish containing a shoot-inducing (SI) medium consisting of Murashige–Skoog (MS) medium (Murashige and Skoog 1962) with 2 mg/L 6-benzylaminopurine (BA), 1 mg/L ABA, and 0.8 % (w/v) agar. The pH was adjusted to 5.7–5.8 before the addition of agar, and all media were autoclaved at 121 °C for 15 min. Cotyledons were excised from post-germination seedlings (Fig. 1a, b). Cotyledons were first cut in half transversely, and the distal parts were discarded. Then, the proximal parts of the explants were cut into 2 pieces longitudinally (Fig. 1c) and immediately subjected to *Agrobacterium* infection.

### *Agrobacterium* strain and binary vector

The *A*. *tumefaciens* strain EHA105 harboring the binary vectors pIG121-Hm, pIG-sGFP, and pGFP-S65C was used for transformation (Nanasato et al. 2011). *Agrobacterium* was cultured with 20 mL of Luria–Bertani (LB) medium (pH 5.2) containing 10 mM 2-(*N*-morpholino)ethanesulfonic acid (MES), 50 mg/L kanamycin, 25 mg/L chloramphenicol, 25 mg/L rifampicin, and 20 μM of acetosyringone at 28 °C until optical density at 600 nm (OD_600_) of 0.4–0.8 was achieved. The *Agrobacterium* culture was centrifuged and resuspended in an inoculation (IN) medium containing SI medium buffered with 10 mM MES to pH 5.2. Varying concentrations (0, 50, 100, 200, and 500 μM) of acetosyringone were added to the IN medium, and the final concentration of *Agrobacterium* (measured by OD_600_) was adjusted to 0.01, 0.1, 0.5, or 1.0. Prior to inoculation, the resuspended *Agrobacterium* inoculum was gently shaken at 28 °C for about 2 h for efficient induction of *vir* genes (Hiei et al. 1997).

### Inoculation, co-cultivation with *Agrobacterium*, selection, and regeneration of transgenic plants

**Fig. 2:**
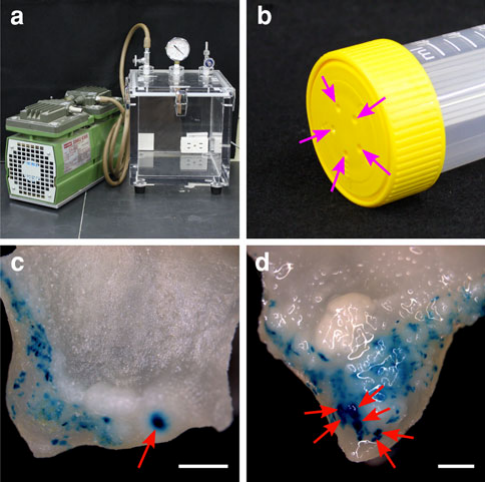
Vacuum infiltration for improvement of infection efficiency. **a** A vacuum system consisting of a vacuum pump (*left*) and desiccator (*right*). **b** A 50-mL bioreactor tube with the cap having an in-built 0.22-μm hydrophobic membrane. *Purple arrows* indicate holes for gas exchange. **c**,** d** GUS activity in proximal regions of explants with immersion (**c**) and vacuum infiltration (**d**) of *Agrobacterium*. *Red arrows* indicate GUS-positive cell clusters. *Bars* 1 mm

Dissected explants were immersed in the *Agrobacterium* inoculum for 10 min. For vacuum infiltration treatment, a vacuum system was constructed (Fig. 2a) as follows: a vacuum pump (DA-60D; ULVAC, Kanagawa, Japan) was connected to a desiccator (VM-C; AS ONE, Osaka, Japan). Dissected explants were placed in a bioreactor tube (TPP TubeSpin; 50 mL; TPP Cell Culture Plastics, St. Louis, MO, USA; Fig. 2b) containing 20 mL of the *Agrobacterium* inoculum, and 2 sessions of vacuum infiltration were applied for 5 min at −0.094 MPa in the desiccator. The vacuum was relieved slowly to prevent damage from sudden pressure change. Excess *Agrobacterium* suspension was removed using sterilized filter paper. For determining a culture support suitable for co-cultivation, infected explants were placed in a plastic 9-cm Petri dish containing IN medium with 3 kinds of culture support: 40 mL of 0.8 % agar gel (method I), 40 mL of 0.8 % agar gel overlaid with a piece of sterilized filter paper (method II), and 3 pieces of sterilized filter paper moistened with 5.5 mL of IN medium (method III), following a previous report (Ozawa 2009). These dishes were sealed with Parafilm (Pechiney Plastic Packaging, Chicago, IL, USA) and placed in the dark at 25 °C for 3 days. After co-cultivation, explants were washed 5 times with sterilized distilled water, blotted dry, and then transferred to a selection medium containing SI agar medium with 10 mg/L meropenem (Ogawa and Mii 2007) and 50 mg/L kanamycin. Explants were subcultured onto fresh media after 2 weeks. After culturing on the selection medium for 4–6 weeks, the regenerated shoots were excised and transferred to a shoot elongation (SE) medium containing half-strength MS medium with 1 mg/L gibberellin A_3_ (GA_3_), 0.8 % agar, 10 mg/L meropenem, and 50 mg/L kanamycin. Non-chimeric transgenic lines were selected via axillary bud culture in SE medium. Rooting plants were acclimatized and grown in a closed greenhouse at day/night temperatures of 28 °C/23 °C.

### Visible marker assay

Histochemical β-glucuronidase (GUS) assays were performed on cotyledonary explants from the 7th day after the elimination of *Agrobacterium*. The GUS staining procedure was as previously described (Nanasato et al. 2011). The data were analyzed statistically using Tukey’s test.

Green fluorescent protein (GFP) fluorescence from transgenic plants was observed using the Leica MZ16FA epifluorescence stereomicroscope (Leica Microsystems, Wetzlar, Germany) equipped with a light source consisting of a 100-W mercury bulb, a FITC/GFP filter set with a 480-nm excitation filter, and a 510-nm long-pass emission filter producing blue light.

### DNA isolation and polymerase chain reaction analysis

For polymerase chain reaction (PCR) analysis, genomic DNA was extracted from leaves of cucumber plants as described previously (Edwards et al. 1991). Primer pairs used for amplifying synthetic GFP protein gene (*sGFP*) were 5′-ctgggtaccatggtgagcaagggcgaggag-3′ and 5′-gcgactagtttacttgtacagctcgtccat-3′, those for amplifying *HrcA*, distributed in *Agrobacterium* (Nakahigashi et al. 1999), were 5′-catcgtcgaaggttatctcgatacg-3′ and 5′-tataatcgaccatcggtacgatacg-3′, and those for amplifying *Actin* were 5′-aatccagacactgtactttctttc-3′ and 5′-tctaatgaaaatattgactgaacg-3′. PCR amplification was performed as follows: 94 °C for 2 min, 30 cycles of 94 °C for 30 s, 60 °C for 30 s, and 72 °C for 1 min, followed by a final extension of 72 °C for 7 min. PCR products were separated on a 1.5 % agarose gel and visualized by ethidium bromide staining.

### Southern blotting

Genomic DNA was isolated from developing young leaves using DNAs-ici!-P kit (Rizo, Tsukuba, Japan), and RNA was removed with RNase A. Genomic DNA (20 μg) was digested with *Hin*dIII, separated on a 0.7 % agarose gel, and transferred onto nylon membranes, positively charged (Roche Diagnostics, Indianapolis, IN, USA) with 20× saline-sodium citrate (SSC) buffer. A digoxigenin (DIG)-labeled DNA probe specific for the *NPT*II coding sequence was used for Southern hybridization, and detection was performed according to the manufacturer’s instructions (Roche Applied Science, Penzberg, Germany).

## Results and discussion

### Culture of cotyledonary explants

We prepared cotyledonary nodes as explants (Fig. 1a–c) from germinated seeds. As in other Cucurbitaceae (Kim et al. 2010; Lee et al. 2003; Nanasato et al. 2011; Tabei et al. 1993), regeneration was observed at the junction of cotyledon and hypocotyl in the proximal parts of cotyledons (data not shown). In addition, water absorption of seeds at higher temperature (28 °C) was effective for assuring uniform and rapid germination of seeds. Incubation at 25 °C often led to insufficient water absorption in seeds (data not shown). ABA has been used to stimulate embryogenic callus (Rai et al. 2011) as well as to stimulate adventitious shoots in various cucumber cultivars (Gal-On et al. 2005; He et al. 2006; Rajagopalan and Perl-Treves 2005; Tabei et al. 1998; Vasudevan et al. 2007). The molecular mechanism of ABA-stimulated shoot induction in cucumber remains unknown. ABA may be involved in the regulation of water content in explants that do not become vitrified and/or may activate stress-tolerance genes, resulting in improving regeneration efficiency. Interestingly, this effect is observed only in cucumber; other Cucurbitaceae, for example *Cucurbita moschata*, display negative effects such as necrosis and growth inhibition of explants (Nanasato et al. 2011).

### Effect of various culture supports on infection efficiency

Unsuitable co-cultivation conditions led to several unfavorable phenomena such as overgrowth of bacteria and/or necrosis of explants, resulting in decreased transformation efficiency. In an effort to optimize co-cultivation conditions, the effect of culture supports was examined, namely method I: 0.8 % agar gel; method II: 0.8 % agar gel overlaid with a piece of filter paper; and method III: 3 filter papers moistened with 5.5 mL of liquid medium. According to Ozawa (2009), the use of 3 pieces of No. 2 filter paper of 9 cm diameter (Advantec, Tokyo, Japan) and 5.5 mL of co-cultivation medium was most suitable for filter paper wick culture. Infection efficiency was estimated by the number of GUS-positive cell clusters, not spots, on the proximal parts of explants. Adventitious shoots from direct organogenesis are of multicellular origin (Norris et al. 1983); therefore, counting cell clusters with GUS is important for efficient evaluation of infection. *Agrobacterium* harboring pIG121-Hm were resuspended to OD_600_ 0.1 in IN medium and explants were immersed in the bacterial suspension for 10 min. After excess suspension was eliminated, different explants were cultured by methods I–III. GUS assay was performed on the explants the 7th day after elimination of *Agrobacterium* with meropenem (Fig. 1e–g). In methods I and II, fewer than 1 % of explants had GUS-positive cell clusters (Fig. 1e, f; Table 1), while in method III, 29 % of explants had GUS-positive cell clusters (Fig. 1g; Table 1). The mean number of GUS-positive clusters per explant in method III was 50 times greater than those of methods I or II. Moreover, explants from method III were obviously healthy and gradually turned green after co-cultivation (Fig. 1d). We evaluated explant health by “greening rate,” which is calculated as the ratio of the number of explants that were green and expanding normally to the total number of explants. The greening rate for method III was about twice those for methods I and II (Table 1). The mean fresh weight for method III was 103.7 mg, or about 3 and 2.6 times the respective weights for methods I and II (Table 1). Ozawa (2009) showed that liquid-medium-moistened filter paper wicks effectively regulated the growth rate of *Agrobacterium* and consequently led to improved cell viability in the transformed callus from rice. We have shown previously that filter paper wicks increased *Agrobacterium* infection efficiency in *C*. *moschata* (Nanasato et al. 2011). These reports and our experimental results support the utility of filter paper wicks for co-cultivation procedures. Method III was thus the most suitable culture support for cucumber transformation.

**Table 1:** Effect of culture support of co-cultivation on *Agrobacterium* infection frequency and growth rate

Culture support^a^	Total no. of explants	Explants with GUS-positive cell clusters (%)	Mean no. of GUS-positive cell clusters per explant	Greening rate (%)^b^	Mean of fresh weight per explant (mg)
Method I	100	0 a	0 c	51.0 e	32.9
Method II	101	1.0 a	0.01 c	57.4 e	39.1
Method III	144	29 b	0.49 d	100 f	103.7

Means within columns followed by the same letter are not significantly different by Tukey’s test at *P* ≤ 0.05. *A*. *tumefaciens* harboring pIG121-Hm was resuspended (OD_600_ 0.1) in IN medium. Cotyledonary explants were immersed in a bacterial suspension for 10 min and co-cultured for 3 days on 3 types of culture support containing IN medium. Co-cultured explants were placed for 7 days in SI-agar medium supplemented with 10 mg/L meropenem, and then GUS-positive clusters, greening rate, and fresh weights of explants were measured

^a^Method I: 0.8 % agar gel; Method II: 0.8 % agar gel overlaid with a filter paper; Method III: 3 filter papers moistened with 5.5 mL of IN medium

^b^Greening rate = (number of green and healthy explants/total number of explants) × 100

### Optimization of acetosyringone concentration for efficient infection in explants

Acetosyringone is known to be a *vir* inducer (Hiei et al. 1997). With the purpose of optimizing the concentration of acetosyringone for efficient transformation, 0, 50, 100, 200, and 500 μM of acetosyringone were added to IN medium and infection efficiency was examined (Table S1). The percentage of explants with GUS-positive clusters increased gradually from 39 to 64.9 % as the concentration of acetosyringone was increased from 0 to 200 μM, whereas 500 μM of acetosyringone decreased the percentage to 49 %. The mean number of GUS-positive clusters per explants was also highest in 200 μM of acetosyringone. Gal-on et al. (2005) used the same concentration (200 μM) of acetosyringone for co-cultivation. In contrast, other groups used lower concentrations: Vasudevan et al. (2007) used 20 μM, and He et al. (2006), Miao et al. (2009), and Nishibayashi et al. (1996) used 100 μM acetosyringone. In our study, even the highest concentration (500 μM) of acetosyringone had no negative effect on explants. In addition, it did not increase the infection efficiency. Accordingly, we selected an acetosyringone concentration of 200 μM for IN medium.

### Increase of infection efficiency by vacuum infiltration with *Agrobacterium* suspension

In an effort to improve the efficiency of *Agrobacterium* infection of the proximal parts of explants, we examined vacuum infiltration of the explants with *Agrobacterium* suspension. Vacuum infiltration enhances transformation efficiency in a wide variety of plant species, including *indica* rice (Lin et al. 2009), citrus (de Oliveira et al. 2009), and cowpea (Bakshi et al. 2011). To maintain aseptic conditions during vacuum infiltration, a bioreactor tube with a cap having holes for gas exchange was used (Fig. 2b). This allowed vacuum infiltration of *Agrobacterium* suspension into explants in the tube without the necessity of loosening the cap. The total time for vacuum infiltration was fixed at 10 min, the same time reported previously for immersion in *Agrobacterium* suspension (Rajagopalan and Perl-Treves 2005; Selvaraj et al. 2010; Tabei et al. 1998). To avert the asphyxiation of explants by vacuum infiltration, the procedure was conducted in 2 sessions. The number of GUS-positive cell clusters in explants was increased 2 times by vacuum infiltration with *Agrobacterium* suspension compared with a single immersion in *Agrobacterium* suspension (Fig. 2c, d; Table 2). This result showed that vacuum infiltration improved the efficiency of *Agrobacterium* infection. We also examined the optimum density of *Agrobacterium* suspension for transformation. Optical densities of 0.01, 0.1, 0.5, and 1.0 at 600 nm were tested (Table 3). Lower densities (OD_600_ 0.01 and 0.1) were not effective for transformation, whereas the highest density (OD_600_ 1.0) decreased infection efficiency. Some researcher studies have used high densities of *Agrobacterium* suspension for infection, such as OD_600_ 0.8 (Miao et al. 2009) and 1.0 (Rajagopalan and Perl-Treves 2005). However, a moderate density (OD_600_ 0.5) was most suitable for efficient transformation. It was thought that a moderate density could contribute to protecting explants from severe damage by *Agrobacterium* and contamination of the regenerated adventitious shoots.

**Table 2:** Effect of vacuum infiltration on *Agrobacterium* infection frequency

Inoculation	No. of explants	Explants with GUS-positive cell clusters		GUS-positive cell clusters	
		*n*	%	*n*	Mean per explant
Immersion for 10 min	117	29	24.8 a	48	0.41 c
Vacuum infiltration for 5 min × 2	117	50	42.7 b	89	0.76 d

*A*. *tumefaciens* harboring pIG121-Hm was resuspended (OD_600_ 0.1) in IN medium supplemented with 200 μM acetosyringone. Cotyledonary explants were inoculated with the bacterial suspension and co-cultured for 3 days on filter paper wicks containing liquid co-cultivation medium. Co-cultured explants were subjected for 7 days to SI-agar medium supplemented with 10 mg/L meropenem, and then GUS staining assay was performed. Means within columns followed by the same letter are not significantly different by Tukey’s test at *P* ≤ 0.05

**Table 3:** Effect of turbidity of bacterial suspension on *Agrobacterium* infection frequency

Density of *Agrobacterium* (OD_600_)	No. of explants	Explants with GUS-positive cell clusters		GUS-positive cell clusters	
		*n*	%	*n*	Mean per explant
0.01	153	24	15.7 a	26	0.17 d
0.10	152	51	33.6 b	77	0.51 e
0.50	154	90	58.4 c	173	1.12 f
1.0	160	68	42.5 b	111	0.69 e

*A*. *tumefaciens* harboring pIG121-Hm was resuspended and adjusted to various densities in IN medium supplemented with 200 μM acetosyringone. Cotyledonary explants were vacuum infiltrated with the bacterial suspension and co-cultured for 3 days on filter paper wicks containing IN medium supplemented with 200 μM acetosyringone. Co-cultured explants were subjected for 7 days to SI-agar medium supplemented with 10 mg/L meropenem, and then GUS staining assay was performed. Means within columns followed by the same letter are not significantly different by Tukey’s test at *P* ≤ 0.05

### Production of transgenic cucumber plants by an optimized method

**Fig. 3:**
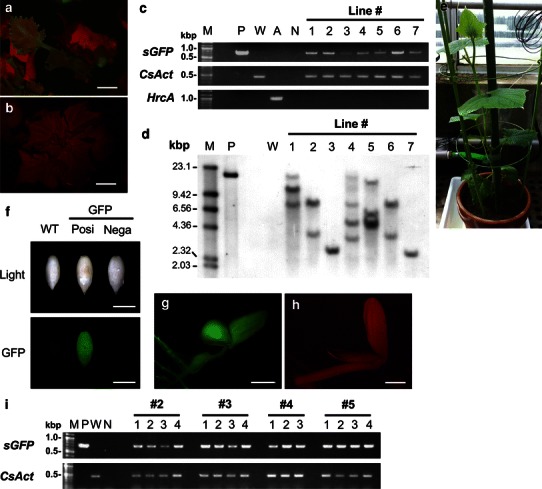
Molecular analysis of transgenic plants. **a** A GFP-positive shoot appearing green under blue light owing to GFP fluorescence. *Bar* 5 mm. **b** A non-transgenic shoot appearing red under blue light owing to autofluorescence of chlorophyll. *Bar* 5 mm. **c** Agarose gel electrophoresis of PCR-amplified DNA from leaf tissue of regenerated shoots. *Lane M* is a 100-bp DNA ladder (NEB); *lane P* is pGFP-S65C (positive control); *lane W* is a wild-type plant; *lane A* is *Agrobacterium* genomic DNA; *Lane N* is without template DNA (negative control); *Lanes 1–7* are different independently regenerated shoots. **d** Southern hybridization analysis of genomic DNA from leaf tissues of selected transgenic plants, vector, and a non-transgenic plant. Genomic DNA (20 μg) from each line was digested with *Hin*dIII, separated on a 0.7 % agarose gel, and transferred onto a nylon membrane. The membrane was hybridized with DIG-labeled *NPT*II probe and detected. *Lane M* is DIG-labeled λ/*Hin*dIII DNA marker (Roche Applied Science); *lane P* is *Hin*dIII digested pIG121-sGFP (positive control); *lane W* is wild-type plant; *lanes 1–7* are different independently regenerated shoots. **e** A transgenic plant growing in a greenhouse. **f** GFP fluorescence of T_1_ seeds. Seed coats were removed for clear observation of GFP fluorescence. *Bars* 5 mm. **g**, **h** A GFP-positive seedling of the T_1_ generation appearing green under blue light owing to GFP fluorescence (**g**) and one of a wild-type plant (**h**). *Bars* 5 mm. **i** Genomic PCR analysis of T_1_ generations. *Lane M* is 100-bp DNA ladder (NEB); *lane P* is pGFP-S65C (positive control); *lane W* is a wild-type plant; *lane N* is without template DNA (negative control)

To evaluate the improvement of transformation efficiency, we attempted to produce *sGFP*-introduced transgenic cucumbers by combination of vacuum infiltration with a moderate density of *Agrobacterium* (OD_600_ 0.5) and co-cultivation on filter paper wicks moistened with 5.5 mL of IN medium supplemented with 200 μM of acetosyringone. *Agrobacterium* harboring pIG-sGFP or pGFP-S65C was infected, and regenerated shoots were screened by kanamycin resistance and GFP fluorescence. Transformed shoots having GFP fluorescence were observed 1–2 months after *Agrobacterium* infection (Fig. 3a), whereas non-transgenic plants showed red chlorophyll autofluorescence (Fig. 3b). The transformed shoots were regenerated along with many adventitious shoots representing escapes. A visual marker such as *sGFP* was useful for identifying transformed shoots among escape shoots, because the regeneration region and the apical meristem were very close and dissection of only the apical meristem was not possible. Transgenic adventitious shoots were transplanted to elongation medium containing GA_3_. GA_3_ effectively stimulated shoot elongation of cucumber, and in many cases roots also developed in the same medium. Occasionally, the detection of GFP fluorescence was difficult in fully expanded mature leaves because of chlorophyll development; however, GFP fluorescence in transgenic roots was easily detected. Repeating axial bud culture several times resulted in the production of non-chimeric transgenic shoots.

To verify gene integration, genomic PCR analysis was performed for regenerated plants. *sGFP* was successfully amplified from seven randomly selected transgenic plants (Fig. 3c). *HrcA* was not amplified from isolated genomic DNA of transgenic plants, suggesting that no *Agrobacterium* contamination occurred in these shoots. To confirm the stable integration of the transgene into the plant genome, Southern blotting was performed using a DIG-labeled *NPT*II probe as described before (Nanasato et al. 2011) (Fig. 3d). The *NPT*II probe was hybridized to digested DNA of transgenic plants and not to that of the wild-type. All selected plants showed different patterns of insertion and the number of signals ranged 1–5. Transformed plants grew normally in the closed greenhouse (Fig. 3e), and T_1_ seeds were obtained by self-pollination. GFP fluorescence was clearly observed in T_1_ seeds by peeling seed coats (Fig. 3f) and in seedlings (Fig. 3g, h). Amplification of *sGFP* and *NPT*II was observed in the T_1_ generations (Fig. 3i). Efficient and stable transformation by our improved method was confirmed. In 4 repeated transformation experiments, the efficiency varied from 7.5 to 16 % with an average of 11.9 ± 3.5 % (Table 4). In transforming cucumber using kanamycin as a selective agent, Selvaraj et al. (2010) reported 7.0 ± 0.23 %, Rajagopalan and Perl-Treves (2005) reported 1.6 %, and Miao et al. (2009) reported 7.1 % transformation efficiency. Our value was one of the highest compared to other previously reported methods.

**Table 4:** Effect of vacuum infiltration for transformation efficiency

Experiment no.	No. of explants^a^	Total no. of transformed plants	Efficiency (%)^b^
#1	35	4	11.4
#2	40	3	7.5
#3	31	5	16.1
#4	40	5	12.5
Total/mean	146	17	11.9 ± 3.5

Mean represents a mean ± SD of 4 independent experiments

^a^Cotyledonary nodes from 1-day-old seedlings were used as explants

^b^Efficiency = (number of shoots with GFP fluorescence and PCR positive/total number of explants) × 100

### Confirmation of inheritance of transgene into T_1_ generation

We examined the segregation pattern of GFP fluorescence in T_1_ seeds derived from line #3 because this line appeared to harbor a single insertion. The *P* value derived from a *χ* ^2^ test using GFP-positive and GFP-negative seeds indicated a good fit to the expected Mendelian ratio of 3:1 at 0.01 % significance (Table 5). These results showed that the transgene was inherited by the next generation.

**Table 5:** Segregation analysis of transgene expression in the T_1_ progeny of the transformant line #3

Total no. of progenies	GFP-positive	GFP-negative	*χ* ^2^ value	*P* value
53	38	15	0.03^a^	0.914

^a^ *χ* ^2^ value indicates a good fit to the expected 3:1 Mendelian ratio at 0.01 % significance

**Fig. 4:**
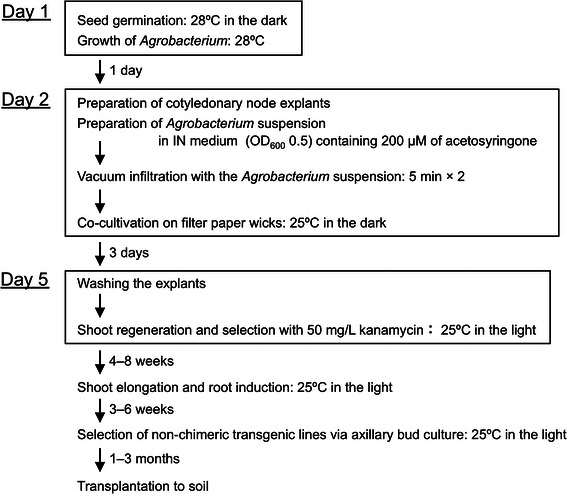
Steps in the transformation of *C*. *sativus* cv. Shinhokusei No. 1 via direct shoot organogenesis from cotyledonary explants

We succeeded in developing an efficient transformation method of Shinhokusei No. 1 (Fig. 4), a prominent variety of cucumber in Japan. Cucumber is still one of the recalcitrant species for transformation, although it has been over 25 years since Trulson et al. (1986) first reported the transformation of cucumber via *A. rhizogenes*. Although *Agrobacterium* infection efficiency is higher in cucumber than in other plant species, it is not sufficient in the proximal region of explants for an effective transformation system. Some groups have tried to improve the infection efficiency by pricking explants with a needle (Rajagopalan and Perl-Treves 2005; Selvaraj et al. 2010; Vasudevan et al. 2007). Although this technique is effective, the efficiency is believed to depend highly on the technician’s skill and to be difficult to reproduce in other laboratories. Because vacuum infiltration, in contrast, is easily repeated under the same conditions, it may contribute to the reproducibility of transformation efficiency. We found that the conditions of co-cultivation were another critical factor in the efficient transformation of cucumber. The use of filter paper wicks in co-cultivation markedly improved the health of explants and increased the number of GUS-positive clusters in the explants compared to other popular culture supports (Fig. 1d; Table 1). Ozawa (2009) reported that filter paper wicks could effectively regulate the growth of *Agrobacterium*. These results suggest that an appropriate *Agrobacterium* cell density during co-cultivation is required for efficient transformation.

While we used kanamycin as a selective agent, other selective agents such as phosphinothricin and mannose have been used for producing transgenic cucumber (He et al. 2006; Selvaraj et al. 2010) and gave transformation efficiencies higher than that of kanamycin. The transformation efficiency of cucumber would be further improved by the use of such selection agents in our improved method.

As described in the introduction, cucumber has received much attention as a model plant for Cucurbitaceae. We propose that the transformation techniques described here will contribute not only to the basic study but also to the molecular breeding of cucumber using various genetic resources.

## Electronic supplementary material

Below is the link to the electronic supplementary material.

Supplementary material 1 (DOCX 9 kb)
