# Supplementary material for: Improvement of Agrobacterium-mediated transformation of cucumber (Cucumis sativus L.) by combination of vacuum infiltration and co-cultivation on filter paper wicks
Source: Plant Biotechnol Rep. 2012 Sep 18;7(3):267–76. doi: 10.1007/s11816-012-0260-1 (PMC3712137; doi:10.1007/s11816-012-0260-1)
Supplement: Supplementary file 1 — Supplementary material 1 (DOCX 9 kb) [file 11816_2012_260_MOESM1_ESM.docx]

**Table S1** Effect of acetosyringone applied to co-cultivation medium on *Agrobacterium* infection frequency

| Acetosyringone (µM) | No. of explants | Explants with GUS-positive cell clusters | | GUS-positive cell clusters | |
| --- | --- | --- | --- | --- | --- |
|  |  | n | % | n | Mean per explant |
| 0 | 105 | 39 | 37.1 a | 53 | 0.5 c |
| 50 | 105 | 51 | 48.6 ab | 87 | 0.83 cd |
| 100 | 99 | 50 | 50.5 ab | 94 | 0.95 cd |
| 200 | 99 | 64 | 64.6 b | 116 | 1.17 d |
| 500 | 98 | 49 | 50.0 ab | 107 | 1.09 de |

*A*. *tumefaciens* harboring pIG121-Hm was resuspended (OD_600_ 0.1) in the co-cultivation medium containing IN medium with different concentrations of acetosyringone. Cotyledonary explants were immersed in the bacterial suspension for 10 min, and then co-cultured on filter paper wicks containing IN medium supplemented with various concentration of acetosyringone for 3 days. Co-cultured explants were placed in SI-agar medium supplemented with 10 mg/L meropenem for 7 days, and then GUS staining assay was performed. Means within columns followed by the same letter are not significantly different by Tukey’s test at *P* ≤ 0.05.
